# Anterior tibial aneurysm involving the transition to the dorsalis pedis, a therapeutic challenge

**DOI:** 10.1590/1677-5449.202401222

**Published:** 2025-05-02

**Authors:** Alexandre Marochi de Castro, Aline Marochi de Castro, Matheus Schimidt Evangelista, Wilson Michaelis, Antônio Lacerda Santos

**Affiliations:** 1 Faculdade Evangélica Mackenzie do Paraná – FEMPAR, Curitiba, PR, Brasil.; 2 Pontifícia Universidade Católica do Paraná – PUC-PR, Curitiba, PR, Brasil.; 3 Universidade Federal do Paraná – UFPR, Curitiba, PR, Brasil.; 4 Hospital Universitário Evangélico Mackenzie, Curitiba, PR, Brasil.

**Keywords:** aneurysm, anterior tibial artery, endovascular procedures

## Abstract

An aneurysm of the anterior tibial artery is always a challenge because it does not provoke symptoms and when a pulsating mass is detected, the aneurysm diameter is already enlarged and there is a greater likelihood of rupture or thrombosis. We report the case of a healthy, 53-year-old, female patient diagnosed with an aneurysm of the anterior tibial artery at the transition to the dorsalis pedis artery, which was identified during ultrasonography performed to investigate plantar fasciitis. The aneurysm was treated by coil embolization and there were no complications.

## INTRODUCTION

An aneurysm is a dilation of an artery to 50% greater than its normal diameter. Aneurysms can involve any artery in the body and if not treated appropriately can cause complications such as rupture, emboli, and thrombosis.^1,2^

There are few reports in the literature of aneurysms involving the tibial arteries in the peripheral territory. The majority of those that do exist are case reports and the condition can thus be considered rare. According to the literature, bilateral aneurysms of the posterior tibial artery may be related to type IV Ehlers-Danlos syndrome. However, the majority of aneurysms in this territory are caused by traumatic events (penetrating or blunt), iatrogenic events, and fractures, or are related to bone tumors. Involvement of the anterior tibial artery is also most often related to traumatic events, normally occurring during sports.^1,3^

The established treatment for these arteries is either artery ligation, if effective collateral circulation is present, or reconstruction with a venous graft, if collateral circulation is insufficient. Treatment can also be accomplished using endovascular techniques such as embolization, thrombin injection, and covered stents.^1,3^

The study protocol was approved by the Ethics Committee at our institution: consolidated opinion number: 7,151,891 and Ethics Appraisal Submission Certificate: 83654524.7.0000.0103.

## PART I – CLINICAL CASE

**Figure 1:**
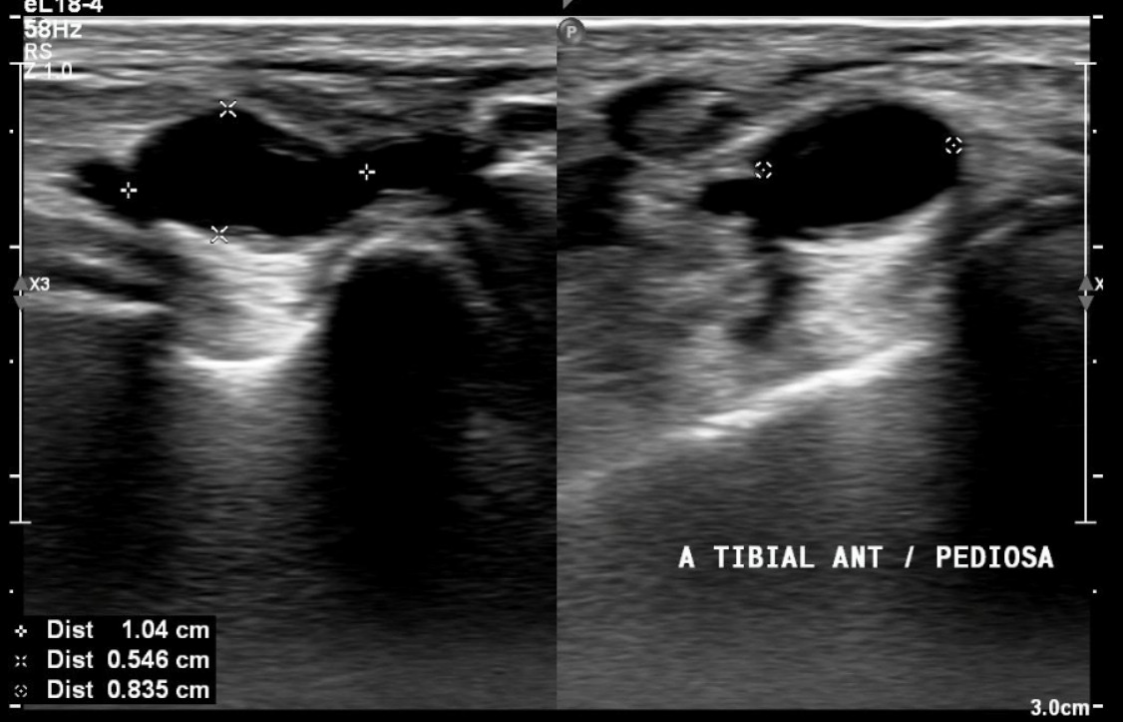
US showing aneurysm of the anterior tibial artery, at the transition to the dorsalis pedis artery. Diameter: 0.835 x 0.546 cm, length: 1.04 cm.

**Figure 2:**
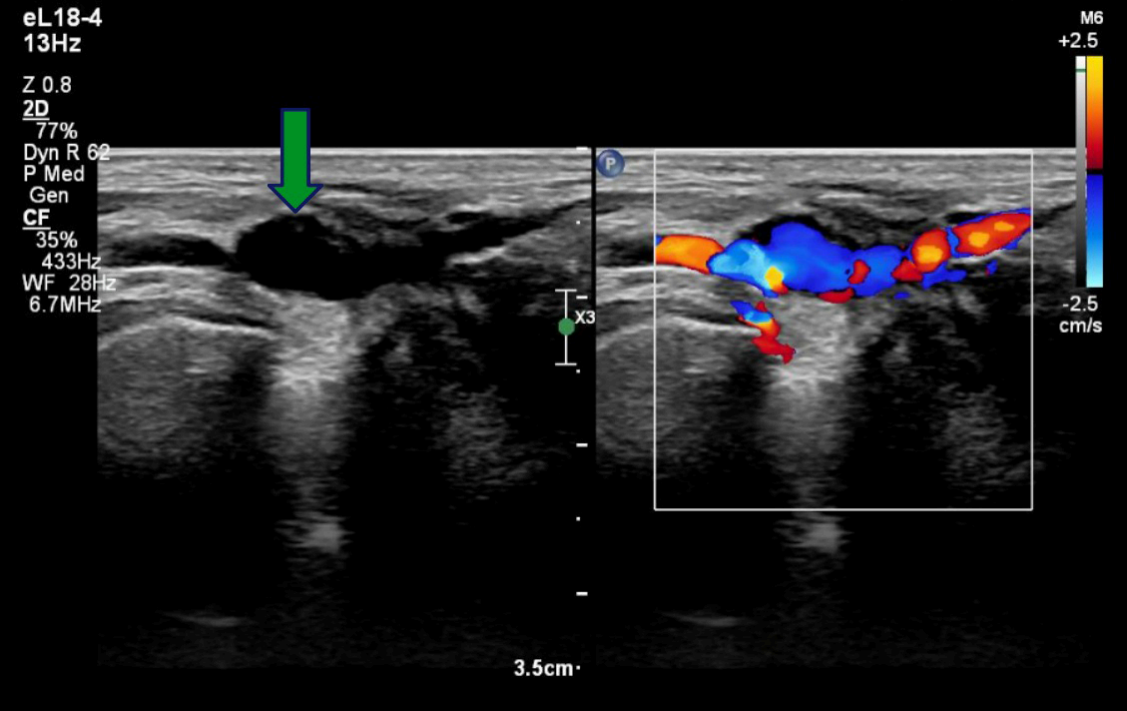
US image (left), showing aneurysmal dilation (at tip of arrow) in the center of the image. Doppler US (right), showing turbulent flow in the region of the aneurysm.

The patient was a 53-year-old female, with no comorbidities, who complained of shooting pains involving the plantar aspect of the left foot, proximal to the calcaneus, with progressively increasing intensity. During the orthopedic investigation, ultrasonography (US) with Doppler confirmed a diagnosis of plantar fasciitis, but also revealed an aneurysm at the transition between the anterior tibial and dorsalis pedis arteries, with a diameter of 0.8 x 0.5 cm and estimated length of 1 cm. (Figures 1 and 2)

**Figure 3:**
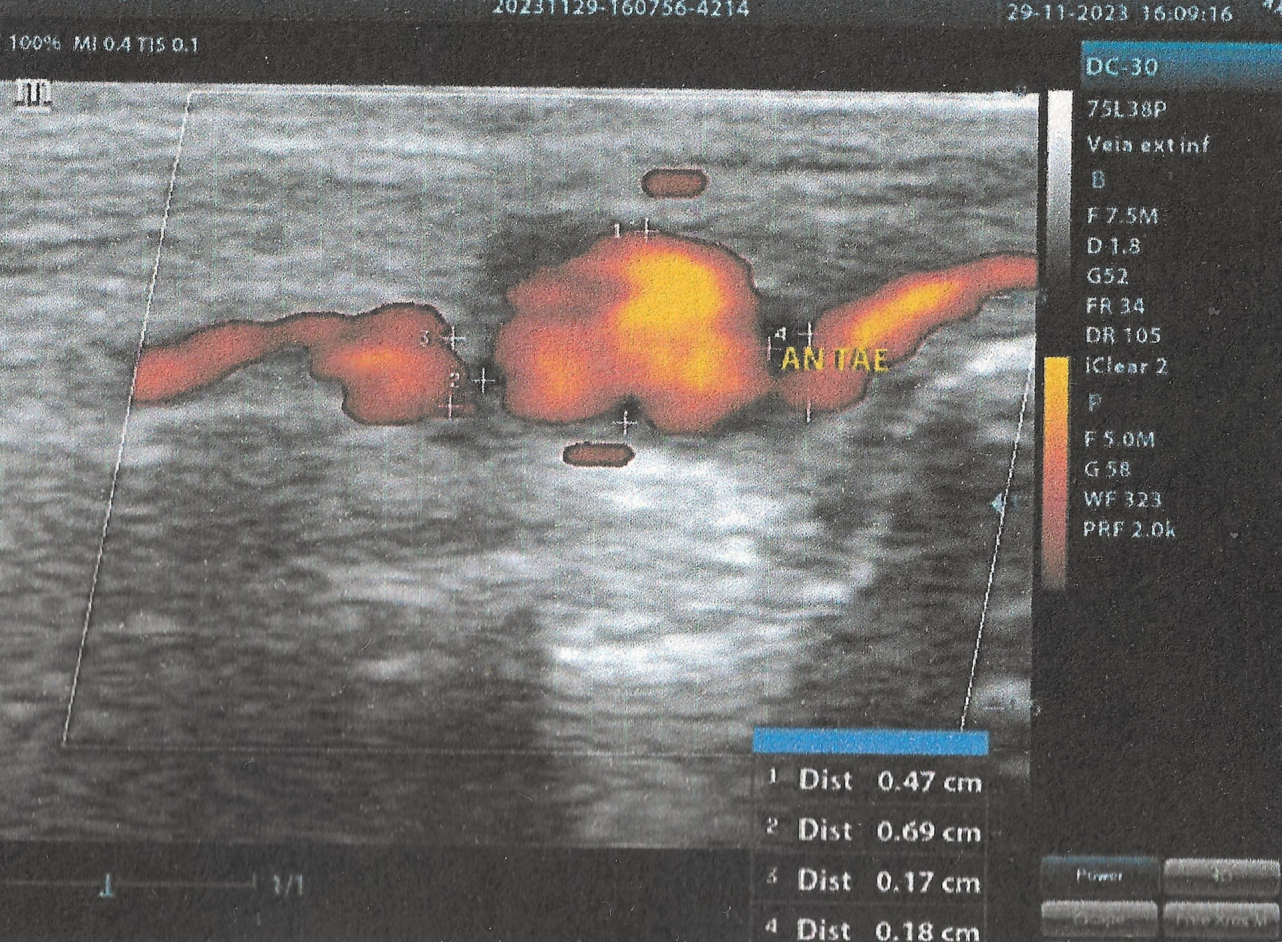
US Doppler scan performed by the vascular surgery team, showing the aneurysm at the transition of the anterior tibial artery to the dorsalis pedis, with turbulent flow in the interior. Size: 0.7 cm side-to-side and 0.5 cm anteroposterior.

In view of these examination findings, the patient was referred to the vascular surgery service. On physical examination, all pulses were palpable: anterior tibial, posterior tibial, and dorsalis pedis, with no obvious signs of the presence of the aneurysm. The patient therefore underwent another Doppler US, confirming the aneurysmal dilation, measuring 0.7 cm in the side-to-side plane and 0.5 cm in the anteroposterior plane, with a 0.17 cm proximal neck and a 0.18 cm distal neck and turbulent flow in its interior (Figure 3).

**Figure 4:**
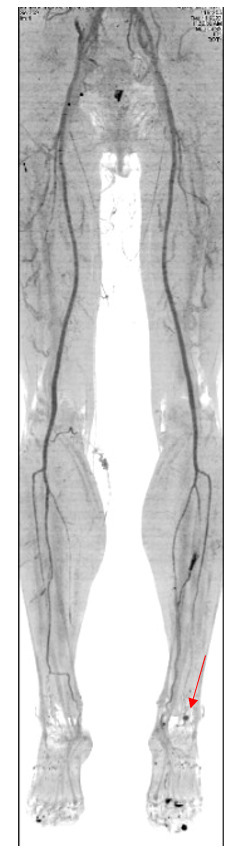
Angiotomography, confirming the aneurysm (red arrow).

**Figure 5:**
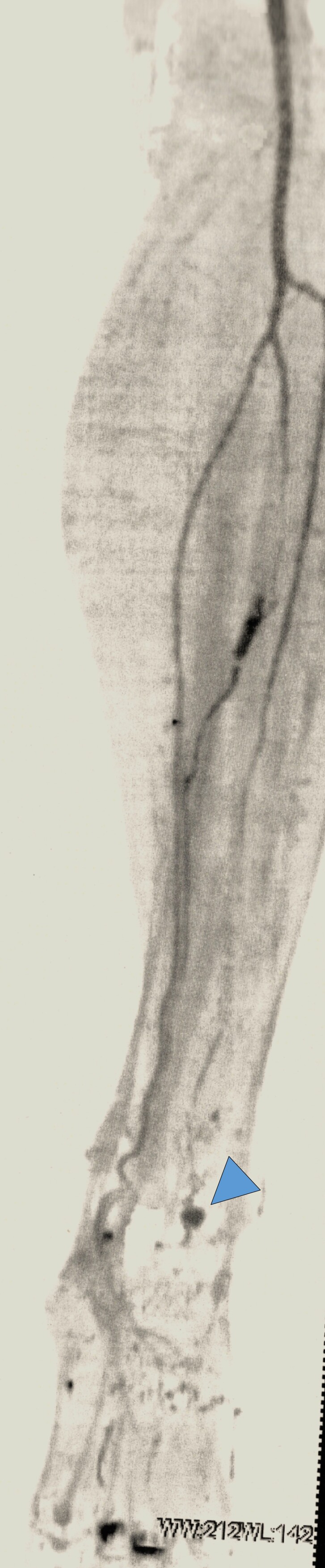
Enlarged angiotomography image, showing the area around the aneurysm (blue arrowhead) in greater detail.

To supplement the diagnosis, angiotomography of the lower limbs was ordered, showing the saccular aneurysm at the transition to the left dorsalis pedis artery, measuring 8.2 x 5.6 mm (Figures 4 and 5). Based on the diagnostic findings, endovascular surgical treatment was indicated.

## PART II – WHAT WAS DONE

**Figure 6:**
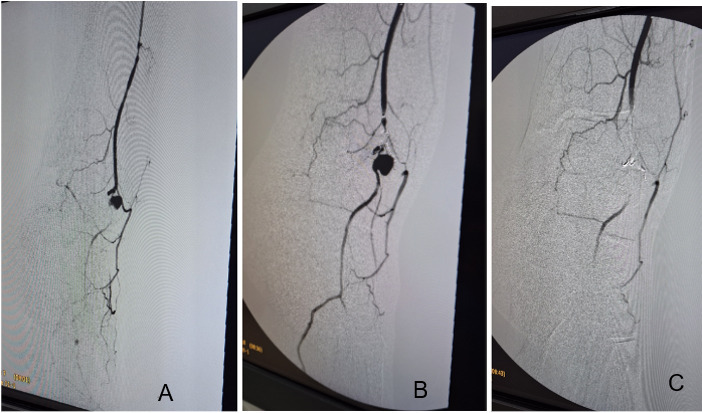
Arteriography. **A** and **B)** Showing the aneurysm as a saccular image at the center, and the adjacent arteries; **C)** post-embolization arteriography, showing the success of the procedure, evidenced by absence of circulation in the aneurysmal region.

The patient underwent arteriography of the left lower limb via anterograde puncture of the left common femoral artery. Once the aneurysm had been identified, it was embolized using an EV3 Echelon™ microcatheter and coils (Figure 6A). A final control arteriography showed that the aneurysmal sac and distal artery were no longer filling, indicating a satisfactory result (Figure 6C). The patient remained in hospital after the procedure and was discharged the following day, free from complications.

One week after the procedure, she attended a follow-up consultation, free from complaints and with good postoperative progress. Physical examination found the left foot well perfused, with palpable anterior and posterior tibial artery pulses and absent dorsalis pedis artery pulse. A US scan of the left lower limb with Doppler showed the anterior tibial artery patent up to the ankle, the aneurysm thrombosed, and retrograde filling of the dorsalis pedis artery via collaterals.

**Figure 7:**
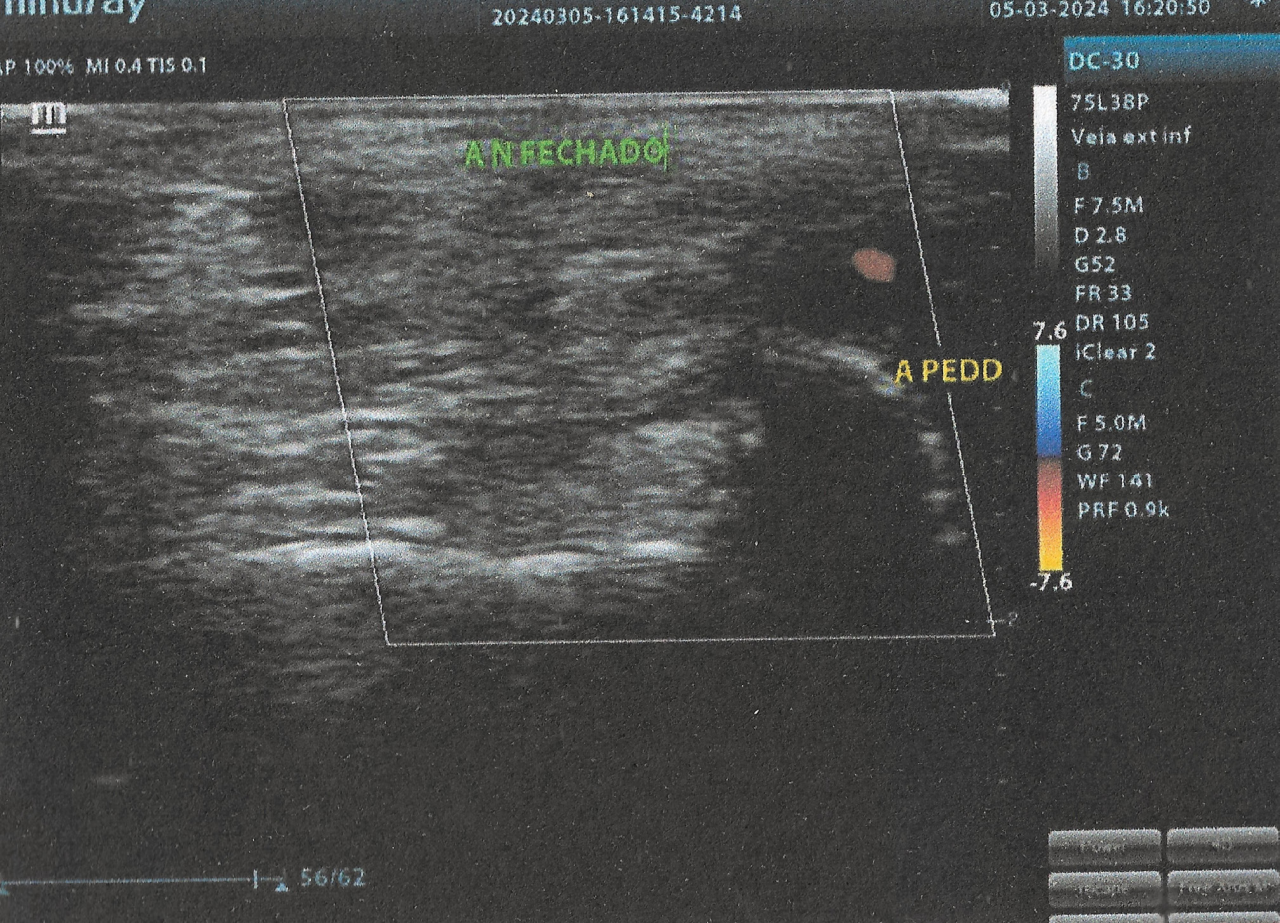
US with Doppler. Aneurysm of approximately 7 mm in size, without flow (CLOSED AN.) and dorsalis pedis artery (D. PED. A.) perfused by retrograde flow from the plantar arch.

Ten months after the surgery, the patient was in outpatient follow-up, stable, and free from trophic ulcers or claudication, revealing good progress. At the last consultation, US with Doppler was conducted, revealing the same findings as the examination conducted 1 week after the procedure (Figure 7).

## DISCUSSION

A Mexican study published by Cárdenas-Guerrero et al.^4^ described the case of a 68-year-old female patient, with hypertension and diabetes, complaining of a tumor on the dorsal aspect of the left foot, with a diameter of 1.5 cm and onset 6 months previously. During the physical examination, he reported pain on palpation of the mobile mass. Open surgical exploration found an aneurysm of the anterior tibial artery.

In a report by Sigterman et al.,^5^ a 59-year-old hypertensive male patient presented with edema of the lateral aspect of the left ankle, without progression. The patient denied prior trauma and reported mild local pain. When examined, a 4 x 5.5 cm pulsating mass was identified. An aneurysm of the anterior tibial was diagnosed with US and angiotomography.

In both studies, the patients exhibited symptoms that prompted them to seek health care, in contrast to our patient, who was asymptomatic and whose aneurysm was identified as an incidental examination finding. One hypothesis that could explain why our patient did not have clinical repercussions was that the aneurysm diameter (0.82 x 0.56 cm) was much smaller than in the cases reported previously.^4,5^

Each of these three studies employed different treatment methods. In our case, an endovascular procedure was performed in conjunction with arteriography, with embolization of the aneurysm. In the Mexican study, the anterior tibial artery was ligated both proximally and distally of the lesion. In the Sigterman et al. case, open surgery was performed, with resection of the aneurysm and reconstruction of the artery using an autologous saphenous vein graft.^4,5^

Although the majority of infrapatellar aneurysms are asymptomatic, symptoms may appear when the aneurysm ruptures, causing limb ischemia, thrombosis, and compression of the fibular nerve. The patient may note pulsating swelling, pain at rest, edema, and claudication, in addition to compartment syndrome, even when there has been no rupture.^4^

A case report by Madison et al.^6^ describes the need for rapid emergency treatment if it is found that aneurysm rupture is imminent. In that study, a 26-year-old patient with type IV Ehlers-Danlos syndrome, possibly the etiology of the aneurysm, felt a sudden “snap” in his leg, followed by pain and the appearance of a pulsating mass in the mid-calf, hitherto never seen. After examinations, an aneurysm of the anterior tibial artery was diagnosed. The patient was referred for surgical treatment, in which the same procedure used for our patient was performed. With the aid of arteriography, coil embolization was performed in the proximal, interior, and distal regions of the aneurysm.^6^

No other Brazilian case reports of anterior tibial artery aneurysm were found. However, there is one case in Brazil of a 49-year-old female patient with an aneurysm of the dorsalis pedis artery. This case is a valid subject for comparison, since our patient’s aneurysm was at the transition from the anterior tibial artery to the dorsalis pedis artery.^7^

The case described was in Aracaju, where the patient presented with a pulsating mass on the dorsal foot, with onset 3 years previously and progressive growth and pain. On ultrasound, the aneurysm measured 1.2 x 1.6 x 2.2 cm. The lesion was ligated proximally and distally.^7^

## CONCLUSIONS

The patient was diagnosed with an aneurysm in the left foot, at the transition from the anterior tibial artery to the dorsalis pedis. She was treated satisfactorily, with coil embolization via endovascular access with the aid of arteriography, providing safe, effective, and minimally invasive resolution, with rapid postoperative recovery.
