# Supplementary material for: Anterior tibial aneurysm involving the transition to the dorsalis pedis, a therapeutic challenge
Source: J Vasc Bras. 2025 May 2;24:e20240122. doi: 10.1590/1677-5449.202401222 (PMC12061359; doi:10.1590/1677-5449.202401222)
Supplement: Translation

# Translation: Aneurisma de artéria tibial anterior na transição com a dorsal do pé: um desafio terapêutico

**DOI:** 10.1590/1677-5449.202401221

**Authors:** Alexandre Marochi de Castro, Aline Marochi de Castro, Matheus Schimidt Evangelista, Wilson Michaelis, Antônio Lacerda Santos

**Affiliations:** 1 Faculdade Evangélica Mackenzie do Paraná – FEMPAR, Curitiba, PR, Brasil.; 2 Pontifícia Universidade Católica do Paraná – PUC-PR, Curitiba, PR, Brasil.; 3 Universidade Federal do Paraná – UFPR, Curitiba, PR, Brasil.; 4 Hospital Universitário Evangélico Mackenzie, Curitiba, PR, Brasil.

**Keywords:** aneurisma, artéria tibial anterior, procedimentos endovasculares

## Abstract

O aneurisma de artéria tibial anterior é sempre um desafio, pois não apresenta sintomas e, quando há a percepção de uma massa pulsátil, o aneurisma já apresenta diâmetro aumentado, com uma maior chance de romper ou de trombosar. Relatamos o caso de uma paciente do sexo feminino, 53 anos, hígida, que apresentou o diagnóstico de aneurisma na artéria tibial anterior na transição com a artéria dorsal do pé, sendo identificado a partir de ultrassonografia realizada para investigação de fascite plantar. O aneurisma foi tratado através de embolização com molas e não apresentou complicações.

## INTRODUÇÃO

Aneurisma é a dilatação arterial superior a 50% do seu diâmetro normal, podendo atingir qualquer artéria do organismo e, caso não tratado adequadamente, apresenta complicações, como rotura, embolia e trombose^1,2^ .

Os aneurismas que atingem as artérias tibiais, território periférico, apresentam poucos relatos na literatura. A maioria dos registros se trata de relatos de caso, sendo considerado, assim, uma condição bem mais rara. Segundo a literatura, aneurismas de tibiais posteriores bilaterais podem estar relacionados à Síndrome de Ehlers-Danlos tipo IV. Entretanto, a maioria dos aneurismas desse território decorre de eventos traumáticos (penetrantes ou contusos), iatrogênicos e fraturas ou está relacionado a tumores ósseos. O acometimento da artéria tibial anterior também está mais relacionado a eventos traumáticos, normalmente resultantes da prática esportiva^1,3^ .

O tratamento estabelecido para essas artérias pode ser a ligadura arterial, na presença de circulação colateral efetiva, ou a reconstituição com ponte venosa, caso a circulação colateral não esteja adequada. Também pode ser realizado por via endovascular, como embolização, injeção de trombina e colocação de *stent* revestido^1,3^ .

O protocolo foi aprovado pelo Comitê de Ética de nossa instituição: número do Parecer Consubstanciado: 7.151.891 e Certificado de Apresentação de Apreciação Ética: 83654524.7.0000.0103.

## PARTE I – SITUAÇÃO CLÍNICA

A paciente era do sexo feminino, 53 anos, sem comorbidades e apresentava dores tipo fisgada na face plantar do pé esquerdo, proximal ao calcâneo, que se intensificaram progressivamente. Durante investigação ortopédica, realizou ultrassonografia (US) com Doppler, que confirmou o diagnóstico de fascite plantar, mas também de um aneurisma na transição entre as artérias tibial anterior e dorsal do pé, com diâmetro de 0,8 x 0,5 cm e extensão longitudinal estimada de 1 cm. (Figuras 1 e 2)

**Figura 1:** Ultrassom mostrando aneurisma de artéria tibial anterior, na transição com a artéria dorsal do pé (pediosa). Tamanho de 0,835 x 0,546 cm e 1,04 cm de extensão longitudinal.

**Figura 2:** Imagem de ultrassom (US) (à esquerda), evidenciando a dilatação do aneurisma (ponta da seta) no centro da imagem. US Doppler (à direita): fluxo turbilhonar na região do aneurisma.

Após o achado de exame, a paciente foi encaminhada ao serviço de cirurgia vascular. No exame físico, percebeu-se que todos os pulsos estavam palpáveis: tibial anterior, tibial posterior e dorsal do pé, sem presença de aneurisma aparente. A paciente, então, foi submetida a nova US com Doppler, confirmando a dilatação aneurismática, medindo 0,7 cm no sentido látero-lateral e 0,5 cm no anteroposterior, além de colo proximal de 0,17 cm e colo distal de 0,18 cm, com fluxo turbilhonar no interior (Figura 3).

**Figura 3:** Ultrassom Doppler mostrando aneurisma na transição da artéria tibial anterior e dorsal do pé, com fluxo turbilhonar no interior, realizado pela equipe da cirurgia vascular. Tamanho: 0,7 cm látero-lateral e 0,5 cm anteroposterior.

Para complementação diagnóstica, foi solicitada uma angiotomografia dos membros inferiores, a qual delimitou o aneurisma sacular na transição com a artéria dorsal do pé esquerdo, medindo 8,2 x 5,6 mm (Figuras 4 e 5). Com base nos achados diagnósticos, foi indicado tratamento cirúrgico endovascular.

**Figura 4:** Angiotomografia, confirmando o aneurisma (seta vermelha).

**Figura 5:** Imagem ampliada da angiotomografia, mostrando com mais detalhes a região do aneurisma (cabeça da seta azul).

## PARTE II - O QUE FOI FEITO

A paciente foi submetida a arteriografia de membro inferior esquerdo através de punção anterógrada de artéria femoral comum esquerda. A partir da identificação do aneurisma, optou-se por embolização utilizando microcateter EV3 Echelon™ e molas (Figura 6A). A arteriografia de controle final evidenciou ausência de enchimento do saco aneurismático e da artéria distal, revelando um resultado satisfatório (Figura 6C). A paciente permaneceu internada após o procedimento, recebendo alta hospitalar no dia seguinte, sem complicações.

**Figura 6:** Arteriografia. **A e B)** Evidencia-se o aneurisma, como uma imagem sacular no centro, e as artérias adjacentes; **C)** arteriografia após a embolização, mostrando o sucesso do procedimento, pela ausência de circulação na região do aneurisma.

Uma semana após o procedimento, retornou em consulta sem queixas e com boa evolução pós-operatória. No exame físico, o pé esquerdo estava bem perfundido, com pulsos da artéria tibial anterior e posterior palpáveis, com ausência de pulso na artéria dorsal do pé. Foi realizada US com Doppler de membro inferior esquerdo, que evidenciou perviedade da artéria tibial anterior até o tornozelo, o aneurisma trombosado e o reenchimento retrógrado da artéria dorsal do pé através de colaterais.

Após 10 meses da cirurgia, a paciente segue em acompanhamento ambulatorial, mantendo-se estável, sem lesões tróficas ou claudicação, apresentando uma boa evolução. Na última consulta, realizou US com Doppler, revelando os mesmos achados do exame realizado uma semana após o procedimento (Figura 7).

**Figura 7:** Ultrassom com Doppler. Aneurisma de aproximadamente 7 mm, sem fluxo (A N FECHADO) e artéria dorsal do pé (A PEDD) perfundida com fluxo retrógado pelo arco plantar.

## DISCUSSÃO

Em um estudo mexicano escrito por Cárdenas-Guerrero et al.,^4^ foi descrito um caso de paciente do sexo feminino, 68 anos, hipertensa e diabética, com queixa de tumoração no dorso do pé esquerdo, com 1,5 cm de diâmetro, e que já evoluía há 6 meses. No exame físico, referia dor à palpação da massa móvel. Após realizada exploração cirúrgica aberta, constatou-se um aneurisma na artéria tibial anterior.

No relato de Sigterman et al.,^5^ o paciente do sexo masculino, 59 anos, hipertenso, iniciou com edema em lateral de tornozelo esquerdo, sem aspecto progressivo. O paciente negava trauma prévio e referia uma leve dor local. Quando examinado, percebeu-se uma massa pulsátil de 4 x 5,5 cm. Ao realizar US e angiotomografia, foi diagnosticado um aneurisma de tibial anterior.

Em ambos os estudos, os pacientes apresentaram sintomas que os levaram a procurar o serviço de saúde, diferentemente da nossa paciente, que era assintomática e teve o aneurisma identificado como um achado de exame. Uma das hipóteses levantadas para nossa paciente não ter repercussões clínicas é que o diâmetro do aneurisma (0,82 x 0,56 cm) era bem menor em comparação aos casos referidos previamente^4,5^ .

Os três estudos elencados apresentaram formas de tratamento diferentes. Em nosso relato, foi realizado procedimento endovascular associado a arteriografia, com embolização do aneurisma. Já no estudo mexicano, foi feita ligadura da artéria tibial anterior, proximal e distal à lesão. No caso de Sigterman et al., a cirurgia foi aberta, com ressecção do aneurisma e reconstrução da artéria com veia safena autóloga^4,5^ .

Apesar da maioria dos aneurismas infrapatelares serem assintomáticos, os sintomas podem aparecer quando acontece ruptura, gerando isquemia do membro, trombose e compressão do nervo fibular. O paciente pode perceber uma tumoração pulsátil, dor ao repouso, edema e claudicação, além de síndrome compartimental, mesmo quando não rompido^4^ .

Um relato de caso realizado por Madison et al.^6^ descreve a necessidade de um tratamento ágil e emergente, caso identificado um aneurisma na iminência de romper. No estudo, um paciente de 26 anos com Síndrome de Ehlers-Danlos tipo IV, possível etiologia do aneurisma, sentiu um “estalo” repentino na perna, acompanhado de dor e do aparecimento de uma massa pulsátil na metade da panturrilha, nunca antes notada. Após exames, diagnosticou-se um aneurisma de artéria tibial anterior. O paciente foi encaminhado para tratamento cirúrgico, no qual foi realizado o mesmo procedimento adotado para nossa paciente. Com o auxílio da arteriografia, houve embolização com molas na região proximal, no interior e distal ao aneurisma^6^ .

Não foi encontrado qualquer relato de caso brasileiro de aneurisma da artéria tibial anterior. Apesar disso, há um caso no país de uma paciente do sexo feminino, 49 anos, com aneurisma na artéria dorsal do pé. O caso é válido como critério de comparação, já que a nossa paciente apresentava o aneurisma na transição da artéria tibial anterior com a artéria dorsal do pé^7^ .

O caso foi descrito em Aracaju, onde a paciente relatou o aparecimento de uma massa pulsátil no dorso do pé, há 3 anos, com crescimento progressivo e dor. A medida do aneurisma no ultrassom era de 1,2 x 1,6 x 2,2 cm. Foi realizada ligadura da porção proximal e distal da lesão^7^ .

## CONCLUSÃO

A paciente apresentava aneurisma à esquerda, na transição da artéria tibial anterior com a dorsal do pé. Foi tratada de forma satisfatória, com embolização por molas via endovascular, auxiliado por arteriografia, promovendo tratamento seguro, eficaz e minimamente invasivo, com uma rápida recuperação pós-operatória.
